# Alpha6beta1 integrin expressed by sperm is determinant in mouse fertilization

**DOI:** 10.1186/1471-213X-7-102

**Published:** 2007-09-12

**Authors:** Virginie Barraud-Lange, Nathalie Naud-Barriant, Line Saffar, Liliane Gattegno, Beatrice Ducot, Anne-Sophie Drillet, Morgane Bomsel, Jean-Philippe Wolf, Ahmed Ziyyat

**Affiliations:** 1 Biologie de la Reproduction, UFR SMBH, Université Paris 13, Bobigny, France. Service d'Histologie-Embryologie-Cytogénétique, Hôpital Jean Verdier (AP-HP), Bondy, France; 2 EA 3410 UFR SMBH, Université Paris 13, Bobigny, France; 3 INSERM-INED Epidémiologie-Démographie-Sciences sociales, CHU Bicêtre, Le Kremlin-Bicêtre, France; 4 Entrée Muqueuse du VIH et Immunité Muqueuse, Département de Biologie Cellulaire, Institut Cochin, Université Paris Descartes, CNRS (UMR 8104), Inserm U56, Paris, France

## Abstract

**Background:**

Based on inhibition tests, the alpha6beta1 integrin was suggested to be a sperm receptor, but further experiments using gene deletion techniques have shown that neither oocyte alpha6, nor beta1 integrin subunits were essential for mouse fertilization.

**Results:**

Using Western blot analysis and immunofluorescence, we showed that the mouse sperm expresses the alpha6beta1 integrin. As for oocyte, binding of GoH3 anti-alpha6 antibody to sperm induces a specific inhibition of sperm fertilizing ability. Comparing zona-intact and zona-free eggs in fusion tests, we showed that the removal of the zona pellucida by acid treatment bypasses fertilizing oocyte alpha6beta1 integrin's function in the adhesion/fusion process.

**Conclusion:**

These findings show that alpha6beta1 integrin is expressed by both gametes and is functional in their membranes interaction. These results and previous reports, about fertilization of alpha6 or beta1 integrin subunits deleted oocytes by wild type sperm, suggest that the presence of alpha6beta1 integrin on one of the two gamete membranes can rescue the fertilization process. This hypothesis is further supported by the exchange of membrane fragments occurring between gametes prior to fusion that we recently reported.

## Background

Sperm-egg interaction is a complex molecular process leading to gamete fusion mediated by a series of molecular interactions. Evidences have been put forward suggesting the involvement of members of the ADAMs, tetraspanins and integrins families in this mechanism. Recently, Izumo, a sperm protein belonging to the immunoglobulin superfamily, was found to be essential for the fusion [1]. Three groups reported that *Cd9*-deficient female mice present a dramatic reduction of their fertility due to a lack of fusion ability of their oocytes [2-4]. We recently showed that double knock-out *Cd9*^-/-^/*Cd81*^-/- ^female mice are completely infertile [5] and that, upon fertilization, CD9 tetraspanin controls α6β1 integrin relocation in patches on the egg membrane [6].

Integrins have also been implicated in sperm-oocyte interaction. Based on several experiments, mouse egg α6β1 integrin was described to function as a receptor of the sperm fertilin β. During further experiments, the function-blocking mAb directed against the α6 integrin subunit, GoH3, turned out to inhibit sperm-oocyte binding, and peptides derived from the sperm fertilin β disintegrin domain sequence bind to α6β1 integrin [7-9]. However, mouse gamete binding and fusion tests using GoH3 mAb gave disparate results, partly according to the technique used for ZP removal [7,10,11]. The binding of sperm to mouse oocytes was not inhibited when ZP had been removed using an acidic treatment [12] and the fertilization rate (FR) of cumulus-intact mouse oocytes was not reduced [11]. Recently, we have shown that GoH3 strongly inhibited human sperm-egg fusion when the ZP had been removed by microdissection [6]. Moreover, oocytes from α6 integrin-deficient mice show normal binding and fusion with sperm, suggesting that the α6 integrin is not essential for gamete binding and fusion [11]. These results suggested that the α6 integrin function could be redundant, possibly with another member of the integrin family also expressed on oocyte membrane. However, according to experiments performed by He *et al*., none of the integrins known to be present on the oocyte are essential for gamete fusion [13].

Major questions about integrins' roles in sperm-oocyte fusion remain unsolved. The possible effect of anti-α6β1 antibodies on mouse sperm functions has never been addressed, whereas expression of α6β1 integrin on human spermatozoa has already been reported. The integrin subunits α4, α5 and α6 have also been detected on normal human sperm using flow cytometry, their expressions being reduced in cases of terato- or oligoasthenoteratozoospermia [14]. The β1 integrin subunit has been detected by histochemistry on basement membrane of the tubuli seminiferi, spermatocytes, spermatids and spermatozoa in human [15]. Furthermore, a positive correlation between the expression of β1 integrin on human spermatozoa and their fertilizing ability has been demonstrated suggesting that sperm integrins may be determinant in egg-sperm recognition and interaction [16,17]. Hence, Reddy *et al*. proposed α6β1 integrin as a clinical marker to evaluate sperm quality [16]. Several other studies performed on human sperm demonstrated correlations between their level of integrin expression and their fertilization ability [18-20]. The presence of sperm integrin in human raises the question of its possible expression by mouse sperm. In spite of numerous experiments involving the anti-α6 mAb GoH3 in mouse gamete interaction, this point has never been investigated and discussed, whereas it can account for some of the reported data discrepancies.

Another point deals with the model of ZP-free oocyte that was most frequently used to study gamete interaction [7,9,21]. To focus the studies on events that occur at the plasma membrane level, the ZP was removed by chemical, enzymatic or mechanical methods. Indeed, this model prevents any interference between both gamete membranes and is considered to be more convenient for studying their interaction. However, ZP removal dramatically modifies the local conditions of gamete interaction and may also explain some contradictory results that have been previously reported. We recently showed that upon ZP removal, several integrin subunits, including α6, and tetraspanins are relocated and clustered into patches [6]. Simultaneously, several oocyte functions related to fusion are modified. Hence, in human oocyte, the mAb anti-CD9 is effective in inhibiting gamete fusion only if it is introduced in the medium before zona removal and not after [6], suggesting that CD9 tetraspanin function is bypassed by this membrane remodeling. In this study, we now demonstrate the expression of α6 and β1 integrin subunits by mouse sperm. We performed IVF assay after preincubation of sperm and/or oocyte with antibodies. Experiments were performed using both acid Tyrode (AT) ZP-free and cumulus-intact oocytes. Thus, we demonstrated that mouse sperm express α6β1 integrin and that this integrin is involved in the sperm-oocyte interaction process.

## Results

### Alpha6 and beta1 integrin subunits are expressed on mouse sperm membrane

#### Western blot analysis

**Figure 1:**
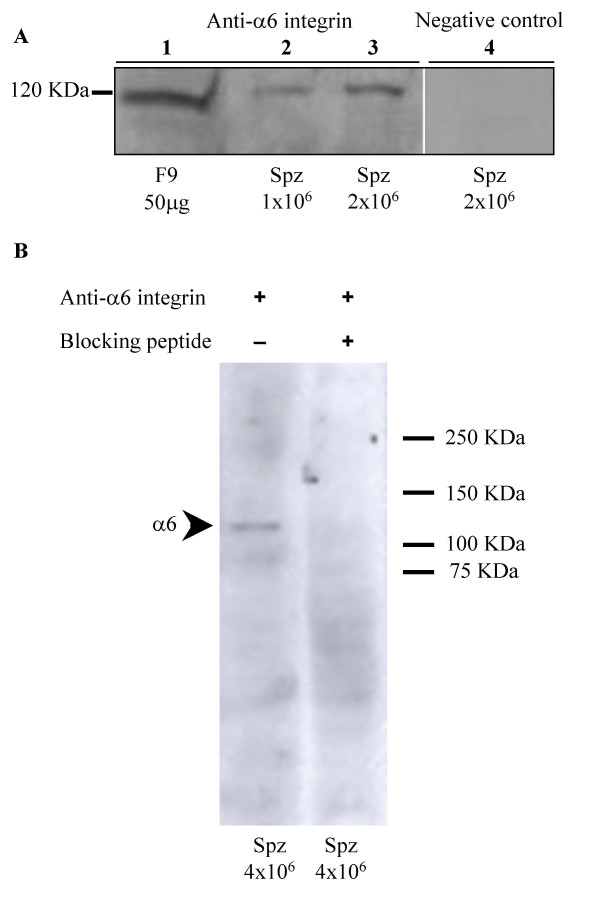
**Expression of alpha6beta1 integrin on mouse sperm**. Alpha 6 integrin subunit expression was investigated by Western blot analysis. Proteins from epididymal sperm were extracted with 1% NP40, subjected to SDS-PAGE under reducing conditions and blotted with two different anti-α6 integrin antibodies to reinforce the specificity of the detection. **A: **120 kDa specific band was detected on sperm extracts after rabbit polyclonal antibody incubation (H-87 at 0,2 μg/ml), (lanes 2 and 3: 1 and 2 millions of sperm, respectively). This band corresponds to α6 integrin subunit as confirmed by the use of the F9 Whole Cell Lysate as positive control (lane 1: 50 μg of proteins). No specific band appeared when a non specific rabbit IgG was used (lane 4). **B**: Using the N-19, goat polyclonal antibody rose against a peptide mapping at N-terminus of α6 integrin subunit, a similar band was detected (lane 1). This band disappeared when the blocking peptide was added (lane 2).

Expression of α6 integrin subunit on cauda epididymal sperm was evaluated by immunoblot analysis. As shown on figure 1A, a specific band at 120 kDa, the expected molecular weight of α6 integrin subunit under reducing conditions, was detected on sperm (lane 2: 1 million and lane 3: 2 millions of sperm) and on F9 Whole Cell Lysate (lane 1: 50 μg proteins), serving as positive control. The control using a non-specific rabbit polyclonal antibody was negative. The specificity of this result has been confirmed using another antibody raised against α6 integrin subunit and its specific blocking peptide. As illustrated in figure 1B, the specific band present at 120 kDa with the anti-α6 integrin antibody (lane 1) has disappeared when the anti-α6 integrin antibody was preincubated with its specific blocking peptide (lane 2). These results demonstrated the expression of α6 integrin subunit on sperm.

#### Immunofluorescence analysis

**Figure 2:**
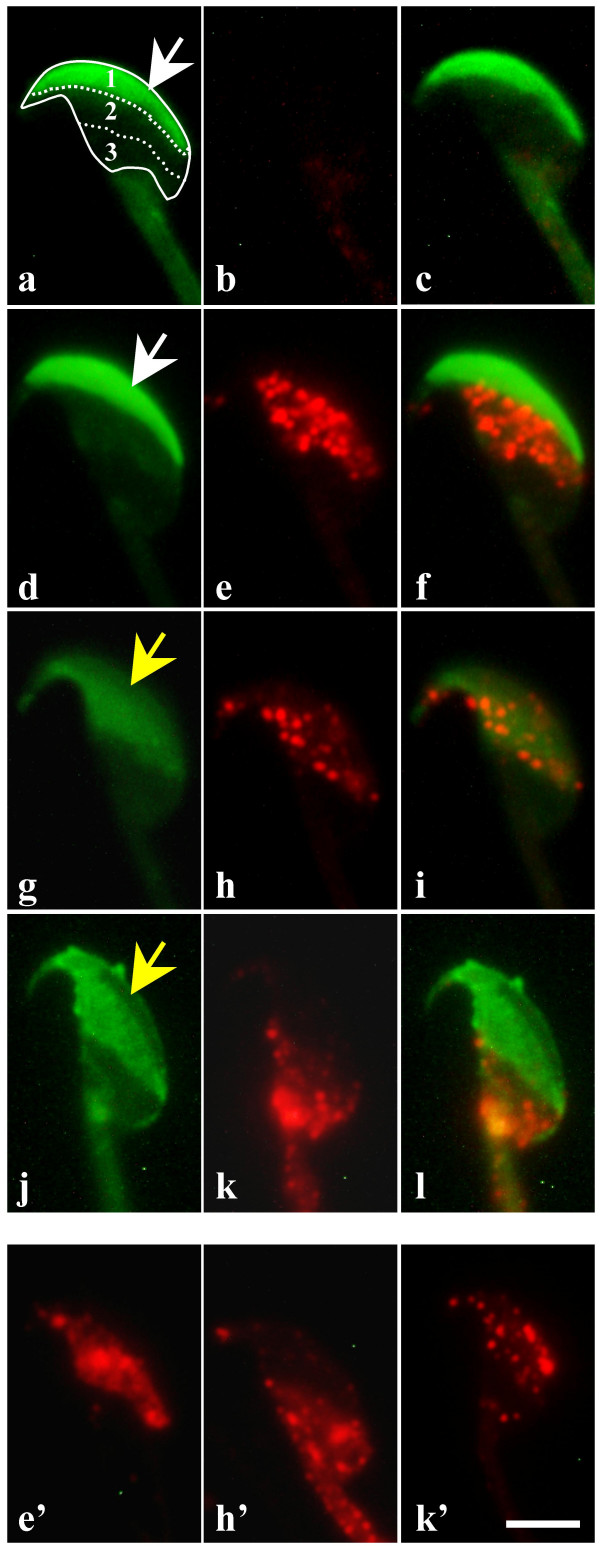
**Expression and distribution of alpha6beta1 integrin on mouse sperm**. Distribution of α6β1 integrin was studied by immunofluorescence microscopy. Spermatozoa were exposed to FITC-PSA (a, d, g, j) after anti-α6 integrin GoH3 antibody (b, e, h, k) or anti-β1 integrin MB1.2 antibody (e', h', k') followed by Alexa Fluor^® ^594 (red) goat anti-rat IgG secondary antibody. The following fluorescent patterns were recorded: negative non capacitated sperm (b), equatorial localization on capacitated sperm (e, e', h, h') and post-acrosomal localization (k, k') only on capacitated AR sperm. c, f, i and l represent composite images generated by superimposition of the green and red signals. Arrows indicate the position of intact (white) or reacted (yellow) acrosome. In order to distinguish the different spermatic regions, they were delimited on the image (a); 1: acrosome, 2: equatorial region and 3: post-acrosomal region. Bar = 5 μm.

Immunofluorescence analysis of mouse sperm was carried out with anti-α6 and anti-β1 integrin antibodies (GoH3 and MB1.2, respectively). In parallel, acrosomal status was assessed by *Pisum sativum agglutinin *(PSA) staining protocol. Data revealed different distribution patterns of the integrin subunits on the sperm membrane as shown in figure 2. Freshly recovered non capacitated sperm did not show any distinct fluorescence (Fig. 2c). After 90 minutes under capacitating conditions, more than 70% of sperm presented integrin molecules on their membranes organized in various patterns: dense fluorescent dots of the equatorial region of non acrosome reacted (AR) capacitated sperm (40%; Fig. 2f) or AR sperm (50%; Fig. 2i) and an exclusive distribution in the post acrosomal region of AR sperm (10%; Fig. 2l). Control isotype or secondary antibody alone gave no signal (data not shown). Similar distribution, staining and relative proportions were found for β1 integrin subunit (Fig. 2e'h'k').

#### Flow cytometry analysis

**Figure 3:**
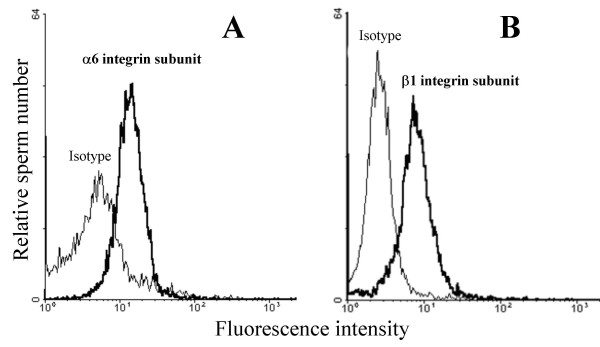
**Flow cytometric analysis of alpha6beta1 integrin expression on mouse sperm**. Expression of α6 and β1 integrin subunits on capacitated mouse sperm was evaluated by flow cytometry. Sperm cells were incubated with the mAb anti-α6 (GoH3) (**A**) or anti-β1 (MB1.2) (**B**) or Rat IgG2a isotype negative control (**A**, **B**), stained with Alexa Fluor^® ^594 (red) goat anti-rat IgG, then analyzed by flow cytometry on the basis of FSC/FL1 (cell size/relative fluorescence) parameters.

Presence of α6 and β1 integrin subunits on capacitated mouse sperm was confirmed afterwards by flow cytometry after indirect immunofluorescence staining. Capacitated spermatozoa labeled with anti-α6 (Fig. 3A) and anti-β1 antibodies (Fig. 3B) were distributed in single peaks with a mean fluorescence intensity of 21 and 34 versus 5 and 8 for control isotype, respectively. Freshly recovered non capacitated sperm was not stained (data not shown).

### Effects of antibodies against alpha6 and beta1 integrin subunits on mouse sperm-egg fusion

Since the presence of α6 and β1 integrin subunits on mouse sperm was demonstrated, we next investigated their involvement during fertilization. We evaluated, in the presence or in the absence of antibodies, the percentage of fertilized eggs (FR) in both cumulus-intact and acid Tyrode (AT) zona-free egg assays and the average number of sperm fused per egg (fertilization index: FI) in AT zona-free egg assays. As these integrin subunits are also expressed by the oocytes [7], we determined to what extent each gamete is involved in the inhibition process. Different groups were studied. The group 1 was the control IVF. In groups 2 and 3, only one of the two gametes (oocytes or sperm, respectively) was preincubated with function-blocking antibodies, washed and inseminated without antibodies. In group 4, both gametes were separately preincubated with the function-blocking antibodies, washed and then inseminated without any antibody as in previous groups. In group 5, oocytes were inseminated in presence of antibodies without any preincubation. In groups 6, 7 and 8, anti-β1 antibody was present during the insemination while oocytes, sperm or both had been preincubated with it. Results differed according to the presence or the absence of ZP. When experiments were performed with isotype (rat IgG2a) or with medium containing sodium azide at 0,02% final concentration as controls, no inhibition was detected (data not shown). It is important to note that none of the function blocking antibody used altered the sperm motility.

#### Effect of GoH3 on cumulus-intact egg insemination (Table 1)

**Table 1:** Effects of alpha6 and beta1 integrin subunits antibodies on sperm-egg fusion

Groups	Antibodies preincubation		Antibodies during IVF	FR (%) cumulus-intact (*n*. oocytes)	FI* AT zona-free (*n*. oocytes)
	Sperm	Oocytes			
**Anti-α6 (GoH3) **at 200 μg/ml					

1	-	-	-	91 ± 3 (85)	4,6 ± 0,3 (51)^e^
2	-	+	-	68 ± 6 (64)^a^	4,8 ± 0,3 (49)^e^
3	+	-	-	50 ± 6 (81)^b,*c*^	2,5 ± 0,2 (44)^b,*e*^
4	+	+	-	31 ± 7 (44)^b^	2,6 ± 0,2 (34)^b,*e*^
5	-	-	+	10 ± 3 (77)^b,d^	4 ± 0,3 (38)^e^

**Anti-β1 (L16) **at 50 μg/ml					

1	-	-	-	72 ± 5 (82)	4,5 ± 0,2 (56)^e^
5-6-7-8^§^	±	±	+	20 ± 5 (269)^b^	2,6 ± 0,2 (108)^e^

FR, fertilization rate; FI, fertilization index; Ab, antibody; AT, acid Tyrode; *, Number of spermatozoa per oocyte;^§^, In groups 2 to 4 of the anti-β1 tests, the antibody was absent during insemination and FR and FI were comparable to the control (group 1) (data not shown), FR and FI in groups 5 to 8 were no significantly different between them and values given here are means; a, significantly different from control, P < 0.0002; b, significantly different from control, P < 0.0001; c, significantly different from group 2, P < 0.03; d, significantly different from group 4, P < 0.003; e, FR on AT zona-free assay was 100%; Results represent the mean ± s. e. m from three different experiments.

Control FR was 91% ± 3. Our protocols of selective preincubation of each gamete allowed us to discriminate between GoH3 effects on oocytes and sperm respectively. When oocytes (group 2) and sperm (group 3) were preincubated with GoH3, the FR was reduced by 26% (68% versus 91%; P < 0.0002) or 45% (50% versus 91%; P < 0.0001), respectively. GoH3 was even more potent in inhibiting sperm than oocyte functions (P < 0.03). When both gametes were preincubated (group 4), the FR inhibition was 65% (31% versus 91%; P < 0.0001) corresponding to the additive effect of GoH3 on each gamete. The three hours of cumulus-intact oocytes insemination with GoH3 and without any preincubation (group 5) gave nearly a 90% inhibition of the FR, compared to the control (10% versus 91%; P < 0.0001). This inhibition was stronger (P < 0.003) than that recorded after preincubation of both gametes with the antibody. This could be explained by the inhibition of lately recruited GoH3 binding sites during the incubation.

#### Effect of G0H3 on acid Tyrode zona-free egg insemination (Table 1)

In all groups of AT zona-free egg assay, membrane gamete interaction is immediate. Because of this extremely rapid process, all eggs were fertilized (FR = 100%). GoH3 mAb had no effect on AT zona-free oocytes since the FI of group 2 was not modified when compared to the control group, showing that ZP removal by AT renders oocytes insensitive to GoH3 effects. Only sperm preincubation (group 3) with GoH3 significantly reduced the FI. Interestingly, if both gametes were inseminated in presence of GoH3, without preincubation (group 5), the FI was not reduced. In contrast, if the same experiment was performed with cumulus-intact eggs, an almost complete inhibition was observed. Therefore: 1) the AT zona removal induced a disappearance of the GoH3 effects on the oocytes, 2) sperm GoH3 effect is not immediate and requires a delay to be effective. This explains that sperm preincubation inhibits ZP-intact but not AT-ZP-free oocytes fertilization and suggests that the time to get through the ZP could be a necessary delay for sperm inhibition to appear.

#### Cumulus-intact and acid Tyrode zona-free egg assays with anti-beta1 integrin subunit antibody (Table 1)

Gamete preincubation with anti-β1 mAb (L16), both in cumulus-intact and AT ZP-free assays, had no effect (groups 2 to 4, data not shown) while the presence of the antibody during insemination impaired gamete interaction (groups 5–8) and was not modified by any preincubation. In cumulus-intact eggs assay, in presence of anti-integrin subunit β1 mAb, the FR was significantly decreased by approximately 70% (P < 0.0001), compared to the control group. In AT zona-free egg assays, all inseminated oocytes were fertilized (FR = 100%), but a significant reduction in the FI (approximately 50%, P < 0.0001) was obtained in all groups.

### Zona pellucida removal modifies alpha6 and beta1 integrin subunits distribution

**Figure 4:**
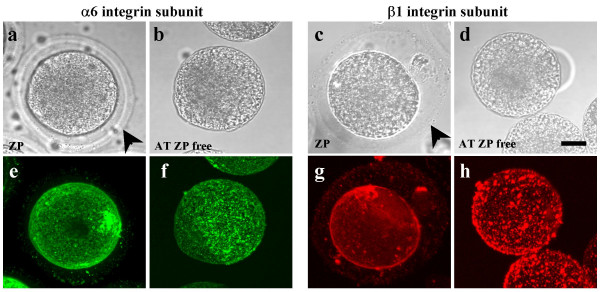
**Acid Tyrode's zona removal modifies distribution of alpha6 and beta1 integrin subunits on mouse oocytes**. Zona-intact or acid Tyrode ZP-free mouse oocytes were incubated with anti-α6 integrin GoH3 (e, f) or anti-β1 integrin MB1.2 antibodies (g, h), then with Alexa Fluor^® ^488 (green) or 594 (red) goat anti-rat IgG respectively and analyzed by confocal microscopy as described in Material and methods. These molecules were distributed homogeneously with fine punctuations around the surface of the zona-intact oocytes (e, g), but both formed patches on the acid Tyrode zona-free oocyte surface (f, h). About 90 sections, representing nearly half of the eggs were collected. The maximum projection function was then used to superimpose the different sections. Transmission images corresponding to each oocyte are shown (a, b, c, and d). Bar = 20 μm.

In an attempt to explain the lack of anti-α6 antibody effect on AT zona-free oocytes, we studied the localization of α6β1 integrin on zona-intact and AT zona-free eggs by immunofluorescence and confocal microscopy analysis. About 90 optical sections, representing nearly half of the eggs, were collected and superimposed using the maximum projection function as shown on figure 4. Whereas the α6 and β1 integrin subunits were distributed homogeneously with fine punctuations around the egg surface of zona-intact oocytes (Fig. 4e and 4g), ZP removal resulted in a modification of the alpha6 and beta1 integrin subunits distribution. Indeed, both formed patches on the AT zona-free oocyte membrane (Fig. 4f and 4h). This integrin redistribution induced upon AT ZP removal seems to put the oocyte at a stage in which integrins are no longer required for adhesion/fusion, explaining in turn the absence of anti-α6 antibody effect on AT zona-free oocytes during fertilization.

## Discussion

Here, we show that the α6β1 integrin is expressed on mouse sperm and is functionally involved in the sperm-oocyte interaction process. These data reinforce the role of α6β1 integrin in the process of gamete interaction [6,7]. We also confirm that AT-ZP removal bypasses the α6β1 integrin function in mouse oocyte at the time of gamete interaction.

We show that mouse sperm express α6β1 integrin by western blot analysis, flow cytometry and immunofluorescence confocal microscopy. This last technique enables to localize the α6β1 integrin on the surface of capacitated and capacitated and AR spermatozoa. Alpha6beta1 integrin clusterisation at the surface of the spermatozoon head in a dotty pattern suggests its activation. As the fertilizing spermatozoon normally present in the perivitelline space is AR, is more likely to present this staining pattern. As the fusion starts at the equatorial and post acrosomal region [22], this localization of the α6β1 sperm integrin reinforces its probable implication in the adhesion/fusion process.

Our results are different from those of other groups [7,23]. In our study, when zona pellucida was totally removed by acid Tyrode, oocytes were insensitive to GoH3 effect. Consequently, we observed gamete interaction inhibition only when sperm was preincubated with GoH3, while Almeida *et al*. reported a total inhibition by GoH3 (after oocyte preincubation) of oocyte-sperm binding and therefore of fusion [7]. This inhibition could have been due to GoH3 effect on both oocyte and sperm. Indeed, in these experiments, very brief chymotrypsin treatment was used and may not have completely removed the zona pellucida [24]. Hence, oocytes were probably not totally insensitive to GoH3 effect. Furthermore, in their experiment, GoH3 have not been washed out from oocyte preincubation medium before sperm addition, contrary to what we did. Therefore, the delay necessary for the spermatozoon to get through the zona pellucida may have stood for the time of GoH3 sperm preincubation. Actually, these experimental conditions were closer to our cumulus-intact IVF ones, accounting for similar results in apparently different IVF conditions. In our previous study, we probably also misinterpreted the GoH3 effect on the human IVF by allotting it exclusively to the oocyte integrin [6]. This effect may have mainly been mediated by the spermatic one.

Two previous studies, using zona-intact or cumulus-intact eggs assays, reported GoH3 lack of inhibition on gamete fusion [11,23]. In the first one, the IVF test was run in small volumes (10 μl). As stated in the report itself, it is known that IVF is inefficient in these conditions (Control FR = 30%). This might have hidden the GoH3 inhibition effect. In the second experiment, contrary to the 3 hours incubation commonly used and as used in our study, insemination lasted 24 hours. Therefore, the evaluation of the FR was not performed according to the presence of decondensed sperm head within the ooplasm, but to the two cell stage criteria. This is not the usual criterion to evaluate fertilization per se, but that of egg activation which can also be achieved by parthenogenetic process.

The most frequent argument to rule out the possible role of α6β1 integrin in gamete interaction is the fertility of α6 or β1 integrin subunits deleted mice oocytes [11,13]. However, all the experiments performed with deleted oocytes, including mating, used wild-type spermatozoa. According to our present results which show the presence of the α6β1 integrin on sperm, these spermatozoa carried the α6 and β1 integrin subunits that had been deleted on the oocyte. This means that, prior to fusion, at the gamete membrane merge, these molecules were present. We therefore have to hypothesize that the presence of this integrin on one of the two fusing membranes is sufficient for fusion. Such hypothesis is supported by the sperm oocyte membrane exchange occurring prior to fusion we reported recently [25]. In this work, we have shown that CD9 tetraspanin is transferred with the exchanged membrane fragment from oocyte to sperm. One can hypothesize that sperm α6β1 integrin could transfer too and, therefore, may have rescued adhesion and fusion in deleted oocytes. The way to definitively answer the question of the implication of α6β1 integrin in gamete interaction during fertilization is the deletion of α6 and/or β1 integrin gene in both gametes. This part of study is under current evaluation.

In our study, AT zona-free eggs were all fertilized whatever the anti-α6 or anti-β1 integrin subunits antibodies used. This could be explained by the direct free access for sperm to oocyte membrane, preventing the effect of the antibody on the sperm. But, importantly, the FI was not affected even when oocytes were preincubated with GoH3 showing that they had become insensitive to its effects. Thus, the oocyte α6β1 integrin is in such a situation that it is no longer needed. As fusion still occurs, we have to conclude that it had been bypassed by the AT ZP removal. This is another reason for the FR being poorly affected by GoH3 on AT ZP-free eggs. Similar bypasses have already been described in human eggs upon mechanical ZP removal [6]. When mouse intact *Cd9*^-/- ^eggs are inseminated, spermatozoa swim into the perivitelline space without binding to the oolemma. On the contrary, large amounts of spermatozoa tightly bind to the oolemma of AT zona-free *Cd9*^-/- ^mouse eggs [4].

To investigate this hypothesis, we studied α6 and β1 integrin subunits distribution on ZP-intact and AT ZP-free oocytes. α6 and β1 integrin subunits were evenly distributed on the surface of zona-intact mouse eggs (Fig. 4e,g). On the oocyte surface of AT ZP-free eggs, these integrins were detected as well, but in larger regularly arranged patches (Fig. 4f,h). The same relocation had been observed in human egg upon ZP removal [6]. We also have reported a relocation of the α6 integrin subunit upon fertilization of mouse zona-intact eggs [6] suggesting that the two phenomena are linked. We can then hypothesize that fertilization and α6β1 integrin redistribution are physiologically linked. Once this relocation has happened, α6β1 integrin has less or no function in the process of gamete binding and fusion. This means that anti-α6 antibodies have few or no effect on AT ZP-free mouse oocytes. For all these reasons and also because is closer to what the physiology should be, experiments using zona-intact eggs have to be preferred to the zona-free oocytes ones.

Takahashi reported an α6β1 integrin clustering at the point of gamete contact on ZP-free oocyte [9]. The presence of α6β1 integrin on the capacitated and AR sperm suggests another non exclusive interpretation. The cluster of α6β1 integrin at the point of gamete contact could also be due to the superimposition of oocyte and sperm α6β1 integrin detection. The fact that α6β1 is not detectable on the surface of fresh sperm may explain why clusterisation was not reported when using non capacitated sperm [9]. In our study, the fertilizing index of AT ZP-free oocytes was reduced only when sperm had been preincubated with GoH3, supporting the existence of a direct effect of this antibody on sperm functions.

## Conclusion

The presence of functional α6β1 integrin on mouse sperm raises the question about the role of this integrin in gamete interaction. It appears as one piece of a multimolecular binding and fusion complex which should be present on both oocyte and sperm membranes. As it can be bypassed by the oocyte membrane reorganization upon ZP removal, we can hypothesize that binding of its ligand to oolemma α6β1 integrin is one of the first steps of gamete membrane interaction process. The only essential molecules that had been recognized so far for sperm-egg binding and fusion are the CD9 tetraspanin on the oocyte [2-4] and on the sperm membrane, the Izumo immunoglobulin [1], which are up to date the unique unilaterally distributed molecules. It could be interesting to study its relation to the sperm α6β1 integrin. A similar interest should be granted to the integrin potential ligands on the sperm.

## Methods

### Antibodies

The function blocking rat mAbs against the integrin α6 (GoH3) or polyclonal goat anti-β1 integrin (L16) subunits were purchased from R&D Systems (France) and Santa Cruz Biotechnology (USA) respectively. For immunofluorescence studies, the rat mAb anti-mouse β1 integrin (clone MB1.2) came from Chemicon International (USA). Secondary antibodies used in immunocytochemistry and flow cytometry experiments were Alexa Fluor^® ^488 (green) or 594 (red) goat anti-rat IgG from Molecular Probes (Invitrogen, France). Rat IgG2a isotype (Serotec, France) was used as negative control. Vectashield mounting medium supplemented with DAPI and FITC-PSA were purchased from Vector Laboratories (USA). For Western blot analysis, the rabbit polyclonal antibody raised against the C-terminus of mouse integrin α6 (H-87), the goat polyclonal antibody rose against a peptide mapping at N-terminus of integrin α6 (N-19) and the specific blocking peptide of N-19 antibody were from Santa Cruz Biotechnology. The non-specific rabbit polyclonal antibody and the biotinylated goat anti-rabbit IgG were from Vector Laboratories; the polyclonal rabbit anti-goat HRP-conjugated was from DakoCytomation (Denmark); the streptavidin HRP-conjugated was from Immunotech (France).

### Gamete preparation and in vitro fertilization

#### Cumulus-intact or acid Tyrode zona-free oocyte preparation

B6CBA F1 female mice (5–8 weeks old) purchased from Charles River Laboratories (France) were superovulated with 5 IU PMSG and 5 IU hCG (Intervet, France) 48 hours apart. Twelve to 14 hours after hCG injection, the animals were sacrificed by cervical dislocation. Cumulus oophorus were collected by tearing the ampulla wall of the oviduct and placed in Whittingham's medium [26] with 3% BSA (Sigma) at 37°C under 5% CO_2 _in air under mineral oil (Sigma). When needed cumulus cells were removed by a brief exposure to hyaluronidase (Sigma) (0.01%). The ZP was then dissolved with acidic Tyrode's (AT) solution (pH 2.5) (Sigma) under visual monitoring. The AT zona-free eggs were rapidly washed five times and directly fixed, or kept at 37°C under 5% CO_2 _in air for 3 hours to recover their fertilization ability.

#### Sperm preparation

Mouse spermatozoa were obtained from the caudae epididymis of B6CBA F1 mice (8 to 13-week-old) and capacitated at 37°C for 90 minutes in a 500 μl drop of Whittingham's medium supplemented with 30 mg/ml BSA, under mineral oil.

#### In vitro fertilization

Cumulus-intact or AT zona-free eggs were inseminated with capacitated spermatozoa for 3 hours in a 100 μl drop of medium. Then, they were washed and directly mounted in Vectashield/DAPI for observation under UV light (Zeiss Axioskop 20 microscope). Were considered fertilized, the oocyte showing fluorescent decondensed sperm head within their cytoplasm. To assess the FI, the number of decondensed sperm head was recorded.

To test the effect of antibodies on the FR and FI, one or both gametes were separately preincubated for 30 minutes in medium supplemented with GoH3 at 200 μg/ml or L16 at 50 μg/ml prior to IVF. Oocytes were then washed and sperm were diluted 1:100 into the final droplet containing the oocytes. To test fusion, oocytes were inseminated at a final sperm concentration of 10^5^/ml for AT zona-free egg assay and 10^6^/ml for cumulus-intact egg assay in a new drop of Whittingham's medium + 3% BSA with or without the antibody.

### Sperm Immunolabeling and Fluorescence Microscopy

Freshly recovered or capacitated spermatozoa were washed in PBS containing 1% BSA, centrifuged at 600 g for 5 minutes and immediately fixed in 4% PFA in PBS-1% BSA at 4°C for 1 hour. After washing, the fixed spermatozoa were incubated in PBS with 20 μg/ml of rat anti-α6 or anti-β1 integrin subunits antibodies (GoH3 and MB1.2, respectively) for 1 hour and then with 10 μg/ml of Alexa Fluor^® ^594 (red) goat anti-rat IgG for 1 hour. Control immunofluorescent studies were performed using isotype (IgG_2a_) as primary antibody or Alexa Fluor^® ^594 goat anti-rat IgG alone.

Alexa Fluor^® ^594 labeled cells were submitted to the PSA-staining protocol for the sequential detection of integrin distribution and acrosomal status. Spermatozoa were treated for 30 minutes in 95% ethanol at 4°C, washed by centrifugation through PBS and stained with FITC-conjugated lectin PSA (25 μg/ml in PBS) for 10 minutes. After repeated washing with double distilled water, a drop of sperm suspension was smeared on slide, air-dried, mounted with Vectashield/DAPI and covered with a coverslip for analysis. Detection was performed using a Zeiss Axiophot epifluorescence microscope and images were digitally acquired with a camera (Coolpix 4500, Nikon).

### Flow cytometry analysis of alpha6 and beta1 integrin subunits on mouse spermatozoa

Freshly recovered and capacitated sperm surface expression of α6 and β1 integrin subunits were evaluated using a FACScan flow cytometer (Becton Dickinson, San Jose, CA, USA) equipped with argon laser (power: 15 mW, wavelength: 488 nm). Flow cytometer was driven with the Cellquest software (Becton Dickinson). The staining procedure was the same as that used for immunofluorescence. Finally, sperm was kept in 1% PFA in PBS at 10^6 ^sperm/ml. For each sample, 5,000 cells were analyzed at a flow rate of 200–300 cells per second using FSC/FL1 (cell size/relative fluorescence) parameters. The fluorescence data were collected using the logarithmic amplifier. Autofluorescence of the sample was measured using sperm that were never exposed to antibodies and negative controls were performed with the isotype and second antibody or second antibody alone.

### Confocal analysis of indirect immunofluorescence oocyte staining

Immunodetection of the integrin subunits was carried out on ZP-intact and AT zona-free eggs. Oocytes were fixed in 2% PFA diluted in PBS-1% BSA for 20 minutes. For detection of the α6 or β1 integrin subunits, oocytes were incubated with the primary antibody GoH3 or MB1.2 (20 μg/ml each one) for 1 hour at room temperature (RT) and then with the suitable secondary antibodies for 1 hour at RT. Controls were prepared by replacing primary antibody by rat IgG2a isotype.

Oocytes were mounted in PBS 1× in Lab-Tek^® ^chambers (Nalge Nunc International, Naperville, USA) and images were captured using a Leica TCS SP2 AOBS confocal microscope. Ninety oocytes sections were collected. The maximum projection function was then used to superimpose the different sections. Image analysis was performed using ImageJ free-software [27].

### Western Blot analysis

Capacitated sperm were washed twice in PBS, the pellet snap-frozen in liquid N2 and stored at -80°C for further use. Sperm aliquots were lysed in 50 mM Tris (pH 8), 150 mM Nacl, 1 mM EDTA, 0,25% sodium desoxycholate and 1% NP40, supplemented with Protease Inhibitor Cocktail (Sigma) for 1 hour on ice, boiled for 5 minutes with an equal volume of 2× NuPAGE^® ^LDS sample buffer (Invitrogen, France) supplemented with 5% β-mercaptoethanol. Semen lysate was gently sonicated. F9 Whole Cell Lysate (Abcam, France) was used as a positive control for the α6 integrin subunit detection. Sample proteins were separated by 4 to 20% SDS-Polyacrilamide gel electrophoresis (Euromedex, France) and electro-transferred to Immobilon-P membranes (Millipore, France). Membranes were blocked overnight with Western Blocking Reagent (Roche, France) prior incubation with appropriate primary antibodies (Rabbit anti-α6 integrin: 0,2 μg/ml, non-specific rabbit IgG: 0,2 μg/ml) for 90 minutes at 37°C followed by biotinylated appropriate secondary antibodies (biotinylated anti-rabbit: 0,2 μg/ml) and HRP-Streptavidin (0,02 μg/ml), each for 1 hour at 37°C. For competition study between anti-α6 integrin antibody (N-19) and its specific blocking peptide, a mixing of equal volume of each of them was prepared and incubated at 4°C overnight. The day after, membranes were incubated with either this mixing or with the anti-α6 integrin antibody (N-19) alone at the dilution 1/200 (1 μg/ml), overnight at 4°C; the membranes were then kept 1 hour at 37°C before an incubation with the secondary rabbit anti-goat antibody HRP-conjugated (1/2000) for 1 hour at RT. HRP activity was revealed by ECL detection kit (Amersham Biosciences, UK) and autoradiography (BioMax Light Film, Kodac). Film exposure was less than 5 minutes.

### Statistical analysis

Statistical analysis was performed using Statview^® ^package. Means were compared by non-paired t test. Differences were considered significant at *P *< 0.05.

## Abbreviations

AR- Acrosome reacted.

AT- Acid Tyrode.

FI- Fertilization index.

FR- Fertilization rate.

IVF- In vitro fertilization.

PSA- *Pisum sativum agglutinin.*

RT- Room temperature.

ZP- Zona pellucida.

## Authors' contributions

VB-L participated in the design of the study, Western blot analysis, confocal analysis and helped to draft the manuscript. NN-B carried out the sperm immunolabeling and fluorescence microscopy and participated to oocytes confocal analysis. LS and LG carried out the flow cytometry analysis. BD performed the statistical analysis. ASD participated in the Western blot analysis. MB participated in the design of the study, the Western blot analysis and redaction of the manuscript. JPW participated in the design of the study and redaction of the manuscript. AZ conceived and coordinated the study, drafted the manuscript, carried out the gamete preparation and in vitro fertilization assays. All authors read and approved the final manuscript.
